# Development and validation of risk prediction models for large for gestational age infants using logistic regression and two machine learning algorithms

**DOI:** 10.1111/1753-0407.13375

**Published:** 2023-03-08

**Authors:** Ning Wang, Haonan Guo, Yingyu Jing, Yifan Zhang, Bo Sun, Xingyan Pan, Huan Chen, Jing Xu, Mengjun Wang, Xi Chen, Lin Song, Wei Cui

**Affiliations:** ^1^ Department of Endocrinology The Second Affiliated Hospital of Xi'an Jiaotong University Xi'an China; ^2^ Department of Endocrinology and Second Department of Geriatrics The First Affiliated Hospital of Xi'an Jiaotong University Xi'an China; ^3^ Department of Physiology and Pathophysiology, School of Basic Medical Sciences Xi'an Jiaotong University Health Science Center Xi'an China; ^4^ Xi'an Jiaotong University Xi'an China; ^5^ Department of Endocrinology Xi'an China; ^6^ Department of Epidemiology and Statistics, School of Public Health, Medical College Zhejiang University Hangzhou China

**Keywords:** gestational diabetes mellitus, heterogeneity, large for gestational age, lipid profile, prediction models, 妊娠期糖尿病, 异质性, 大于胎龄儿, 血脂谱, 预测模型

## Abstract

**Background:**

Large for gestational age (LGA) is one of the adverse outcomes during pregnancy that endangers the life and health of mothers and offspring. We aimed to establish prediction models for LGA at late pregnancy.

**Methods:**

Data were obtained from an established Chinese pregnant women cohort of 1285 pregnant women. LGA was diagnosed as >90th percentile of birth weight distribution of Chinese corresponding to gestational age of the same‐sex newborns. Women with gestational diabetes mellitus (GDM) were classified into three subtypes according to the indexes of insulin sensitivity and insulin secretion. Models were established by logistic regression and decision tree/random forest algorithms, and validated by the data.

**Results:**

A total of 139 newborns were diagnosed as LGA after birth. The area under the curve (AUC) for the training set is 0.760 (95% confidence interval [CI] 0.706–0.815), and 0.748 (95% CI 0.659–0.837) for the internal validation set of the logistic regression model, which consisted of eight commonly used clinical indicators (including lipid profile) and GDM subtypes. For the prediction models established by the two machine learning algorithms, which included all the variables, the training set and the internal validation set had AUCs of 0.813 (95% CI 0.786–0.839) and 0.779 (95% CI 0.735–0.824) for the decision tree model, and 0.854 (95% CI 0.831–0.877) and 0.808 (95% CI 0.766–0.850) for the random forest model.

**Conclusion:**

We established and validated three LGA risk prediction models to screen out the pregnant women with high risk of LGA at the early stage of the third trimester, which showed good prediction power and could guide early prevention strategies.

## Graphical abstract

**Highlights**

- The logistic regression model consisted of seven commonly used clinical indicators (including lipid profile) and gestational diabetes mellitus subtypes, with an area under the curve (AUC) of 0.760 for the training set and 0.748 for the internal validation set.
- The two machine learning algorithms large for gestational age prediction models included all the variables, the decision tree prediction model had AUCs of 0.813 and 0.779 for the training set and the internal validation set. The random forest prediction model showed AUCs of 0.854 and 0.808 for the training set and the internal validation set.

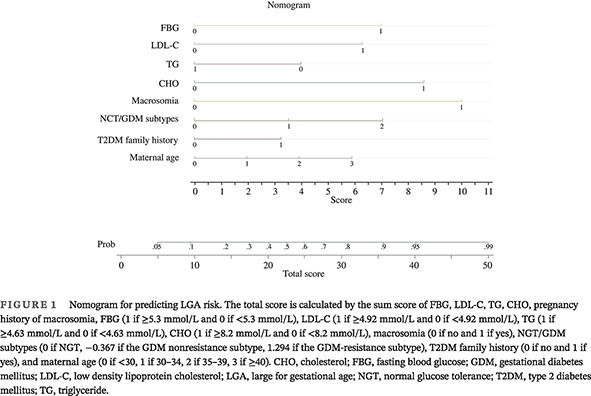

## 摘要

**背景:**大于胎龄儿(Large for gestational age, LGA)是危害母儿生命健康的不良妊娠结局之一。本研究旨在建立妊娠晚期LGA的预测模型。

**方法:**研究对象来自一个1285名中国孕妇队列。LGA诊断为大于同性别新生儿胎龄对应的中国出生体重分布的第90百分位数。根据胰岛素敏感性和胰岛素分泌指标将GDM孕妇分为3型。通过logistic回归和决策树/随机森林算法建立模型, 并通过上述数据进行验证。

**结果:**139例新生儿出生后被诊断为LGA。由7项常用临床指标(包括血脂)和GDM亚型组成的logistic回归模型的训练集AUC为0.760 (95% CI 0.706 ~ 0.815), 内部验证集AUC为0.748 (95% CI 0.659 ~ 0.837)。两种机器学习算法建立的预测模型中, 决策树模型的训练集和内部验证集的AUC分别为0.813 (95%CI 0.786 ~ 0.839)和0.779 (95%CI 0.735 ~ 0.824), 随机森林模型AUC分别为0.854 (95%CI 0.831 ~ 0.877)和0.808 (95%CI 0.766 ~ 0.850)。

**结论:**本研究建立并验证了3种LGA风险预测模型, 可在孕晚期筛选出LGA高风险孕妇, 预测效能较好, 可指导早期预防策略。

## 1 INTRODUCTION

Fetal overgrowth increases the risk of many adverse outcomes for both mother and offspring, such as prolonged labor, cesarean section, shoulder dystocia, admission to the neonatal intensive care unit, and neonatal hypoglycemia after birth, increased risk of obesity and diabetes at childhood and adulthood.^1^, ^2^ Large for gestational age (LGA) is diagnosed as birth weight >90th percentile based on standard growth charts of different population.^3^ Fetuses who were diagnosed as LGA at the second trimester are associated with an increasing risk of LGA at birth.^4^ Fetal overgrowth is directly affected by the nutrients in maternal circulation at the third trimester.^5^ LGA is generally diagnosed before delivery, which leaves a limited phase for early intervention. Therefore, it is necessary to establish LGA prediction models at the early stage of the third trimester, to identify and intervene with pregnant women at high risk of giving birth to LGA offspring, in order to reduce the incidence of LGA at birth.

Gestational diabetes mellitus (GDM) subtypes, which were identified by insulin sensitivity and insulin secretion, may have different mechanisms in causing LGA infants.^6^, ^7^ However, the risk of fetal overgrowth is still increased even after strict glycemic management and in nondiabetic obese mothers.^8^, ^9^ The increased risk of fetal overgrowth in these models may be associated with the increased levels of lipid profile.^10^, ^11^ A meta‐analysis indicates that the abnormal maternal lipid profile, which includes increased triglycerides (TG) and decreased high‐density‐lipoprotein cholesterol (HDL‐C) levels during pregnancy, are associated with the risk of giving birth to LGA for pregnant women.^12^

To our knowledge, there is a limited LGA prediction model that includes GDM subtype as a variable; therefore we aimed to establish a model for early screening and intervention based on GDM subtypes and pregnancy‐related glucolipid metabolism indexes, in combination with other LGA risk factors, which could be used at the early stage of the last trimester to guide prevention strategies.

## 2 METHODS

### 2.1 Data sources

The LGA prediction model was developed based on 1285 pregnant women with relatively complete clinical data during pregnancy from the Department of Obstetrics at the Northwest Women's and Children's Hospital from November 2019 to March 2020. All participants have given their informed consent for participation in this study. We included pregnant women ≥18 years old with full‐term fetuses and relatively complete clinical data at the first and second trimesters for further analysis. Women with prepregnancy type 1 diabetes, type 2 diabetes, or monogenic diabetes were excluded, as were women who met the result of oral glucose tolerance test (OGTT) for fasting blood glucose (FBG) ≥7.1 mmol/L or 2 h plasma glucose ≥11.1 mmol/L, and other metabolic or chronic diseases, including pregnancy complicated with thyroid disease, preeclampsia/hypertension, liver, kidney, or cardiovascular disease. All the pregnant women included in the study did not undergo OGTT at the first trimester and had an FBG <5.1 mmol/L at that period.

### 2.2 Diagnoses and outcome

LGA was diagnosed as >90th percentile of birth weight distribution of Chinese corresponding to gestational age of the same‐sex newborns.^3^ GDM or normal glucose tolerance (NGT) were diagnosed according to the International Diabetes and Pregnancy Study Group (IADPSG) criteria^13^ at the 24th–28th gestational week. GDM subtypes were classified according to the definition by Powe and colleagues on the basis of the distributions on the composite insulin sensitivity index (ISI) and Stumvoll I index of the NGT group (control),^7^ which were calculated by following equations^14^, ^15^ (BMI, body mass index; GLU, glucose; FINS, fasting insulin):

$$\mathrm{ISI}\;\text{composite index}=\frac{10000}{\sqrt{\mathrm{FBG}\times \text{FINS}}\times \left(\text{average}\;\mathrm{GLU}\times \text{average}\;\mathrm{INS}\right)}$$

StumvollIindex=2032+4.681×INS0−135.0×GLU120+0.995×INS120+27.99×BMI−269.1×GLU0

According to Powe's definition,^7^ GDM‐resistance women and GDM‐dysfunction women were defined as below the 25th percentile range of the ISI composite index and the Stumvoll I index based on the distribution of the indexes for the NGT group, respectively. The GDM‐mixed women have the characters of ISI composite index and the Stumvoll I index below the 25th percentile range of the NGT group, and we excluded the women with both ISI composite index and the Stumvoll I index above the 75th percentile range of the NGT group. Because the proportion of LGA in the GDM‐dysfunction and GDM‐mixed subtypes were similar, with no statistically significant difference compared with NGT group, we combined these two groups as the non‐GDM‐resistance group for further analysis.

### 2.3 Definitions and stratification of clinical indexes

Gestational weight gain was calculated as the maternal weight before production minus the prepregnancy weight, while maternal weight before and during pregnancy were recorded by the electronic medical records. Age at menarche was stratified by 11 years old, because current studies found that menarche age <11 years old was a risk factor for metabolic diseases and fetal overgrowth during pregnancy.^16^, ^17^, ^18^ The variables in the model were screened out by the least absolute shrinkage and selection operator (LASSO) regression analysis, the levels of FBG, cholesterol (CHO), TG, HDL‐C, and low‐density‐lipoprotein cholesterol (LDL‐C) at the 28th–32th gestational week were stratified by 5.3 mmol/L, 8.2 mmol/L, 4.63 mmol/L, 1.24 mmol/L, and 4.92 mmol/L, according to the trimester‐specific reference intervals (TSRIs) for blood lipid profiles in Chinese pregnant women.^19^

### 2.4 Data collection and plasma biochemical parameters detection

We made a questionnaire survey to collect the general information of the pregnant women, such as smoking status, alcohol consumption, history of macrosomia, and parity. The plasma biochemical parameters such as lipid profile were detected during the 28th–32th gestational weeks of pregnancy. Standard methods at the certified lab were employed to measure all the laboratory tests, which include glucose oxidase approach for FBG, with an intra‐ and interassay variation factor 2.1% and 2.6%, respectively. The plasma lipid profile, such as TG, total CHO, HDL‐C, and LDL‐C, were tested by the enzyme catalyzed approach. The indexes of the thyroid associated antibodies and insulin were detected by commercially kits (thyroglobulin antibody, TG‐Ab [R‐A‐07‐01, 30‐3000 IU/mL], thyroidperoxidase antibodies, TPOAb [R‐A‐08‐01, 10‐1000 IU/mL], insulin [R‐C‐01‐01, 5–180 mU/mL], 3 V Bioengineering, China). The biochemical indicators of liver, including aspartate transaminase, alanine transaminase, total protein, albumin, globulin, vitamin B12, and ferritin, were detected by the kits that were produced by Shandong 3 V Bioengineering Company, China, and measured by Hitachi 7600 automatic 49 biochemical analyzer.

### 2.5 Statistical analysis

Means ± SD and median (interquartile range) were employed to manifest the normally distributed continuous variables and nonnormally distributed continuous variables, respectively. The former variables were analyzed by the *t* test and the later by Mann–Whitney U test, chi‐square test for the categorical variables when compared two groups. Differences across the four groups (NGT and three GDM subtypes) were compared using one‐way analysis of variance for normally distributed continuous variables, Kruskal–Wallis test for the skewed distributed continuous variables, or chi‐square test for categorical variables. When a *p* < .05, pairwise comparisons between the NGT group and each GDM subgroups were made using the Tukey's test, Dunn's test, or chi‐square test, respectively. *p* values for pairwise comparisons were adjusted using the Bonferroni correction.

### 2.6 Multiple imputation

We conduct multiple imputation using chained equations to replace missing values within 20% of the total, which including vitamin B12, the indexes of plasma lipid profile, and ferritin at the third trimester. We employed five estimation models according to the size of the data and the capacity of the software (Jupyter Notebook 6.4.5, python 3.9.7).

### 2.7 The logistic regression modeling strategies

The data set was randomly assigned by random sample (SPSS 26.0), approximately 70% of all cases for the training set (*n* = 900) and the rest for the validation set (*n* = 385). LASSO regression analysis (STATA 15.0) and the cross‐verification LASSO logistic regression (3 folds, seed 123) with the largest lamda for mean squared prediction error within one SE were conducted to select and determine the variables. Multivariate logistic regression (SPSS 22.0) was employed to modeling the strategies (backward variable selection). We used STATA (version 15.0) to plot the nomograph for generalizing, area under the curve (AUC) to reflect the estimated average optimism of the prediction accuracy, calibration curves to measure the probability of the relationship between the model and observed rate in sets, decision curve analysis (DCA) curves to show the net return, and Hosmer‐Lemeshow test to calculate the differences of the predicted and the true value.

### 2.8 The machine learning (ML) algorithms

We processed the data with Random Under‐Sampling and Synthetic Minority Over‐Sampling Technique (SMOTE) because of the imbalanced quantity of GDM women (402) and NGT women (883); we also used the random undersample to trim most of the classes to reduce the overfitting risk caused by SMOTE. We used decision tree (DT) and random forest (RF) to develop the LGA prediction models by Jupyter Notebook (Anaconda) 6.4.5, and employed Graphviz 2.38 to plot the graphs. DT is made up of a single root node and several internal nodes and leaf nodes, with CART algorithms based on Gini coefficient. RF is an integrate algorithm layered on the top of multiple DT classifiers, which are randomly constructed and controlled by several selected characteristic variables. Bagging and random feature selection were employed to conduct the learning process. We compared the ML models by various approaches: area under the receiver operator characteristic curve, precision, recall, F1‐score, accuracy, and specificity.

## 3 RESULTS

### 3.1 Baseline data

Out of 3829 pregnant women, 1285 women underwent the 75 g OGTT at the 24th–28th gestational week, 402 (10.5%) of whom were diagnosed as GDM according to the IADPSG criteria^13^ and 139 newborns were diagnosed as LGA. Among GDM women, 193 patients (48.0%) were classified into the GDM‐resistance group with the clinical character of insulin resistance (ISI composite 47.56 ± 14.54 vs. 139.14 ± 43.75, *p* < 0.001, Table 1) and insulin hypersecretion (Stumvoll I index 1498.54 ± 301.78 vs. 944.32 ± 244.16, *p* < .001) compared with the NGT group. A total of 138 patients (34.3%) were assigned to the GDM‐dysfunction group with the character of normal insulin sensitivity and decreased insulin secretion (Stumvoll I index 664.45 ± 143.22 vs. 944.32 ± 244.16, *p* < .001), and 71 patients (17.7%) were classified into the GDM‐mixed group with characters of insulin resistance (ISI composite index 80.91 ± 17.34 vs. 139.14 ± 43.75, *p* < .05) and decreased insulin secretion (Stumvoll I index 689.34 ± 188.56 vs. 944.32 ± 244.16, *p* < .001). All women in the three GDM subtypes exhibited an elder maternal age (all *p* < .001), and a higher percentage of family diabetes history (all *p* < .05) compared to the NGT group. Only the GDM‐resistance group showed an elevated pre‐BMI (25.60 ± 2.95 vs. 21.30 ± 2.80, *p* < .001) and infant birth weight relative to the NGT group. For other factors, such as gestational week, smoking status, alcohol consumption, and gestational weight gain, there were no significant differences of the three GDM subtypes compared with the NGT group.

**TABLE 1:** The clinical characteristics of the NGT and GDM subtypes.

	GDM‐resistance	*p* ^a^	GDM‐dysfunction	*p* ^a^	GDM‐mixed	*p*	NGT
Number (*n*)	193		138		71		883
Maternal age (years)	31.77 ± 4.52	< .001	31.65 ± 3.61	< .001	32.44 ± 3.83	< .001	30.27 ± 3.69
Family history of diabetes mellitus (*n*, %)	36 (18.6)	< .001	20 (14.4)	.007	10 (14.1)	.026	48 (5.4)
Pre‐BMI (kg/m^2^)	25.60 ± 2.95	< .001	20.80 ± 2.00	—	20.95 ± 1.66	—	21.30 ± 2.80
Gestational week (weeks)	38.78 ± 1.41	—	38.99 ± 1.24	—	38.94 ± 1.17	—	39.00 ± 1.23
Smoking status (*n*, %)	5 (2.6)	—	0 (0)	—	2 (2.8)	—	19 (2.1)
Alcohol consumption (*n*, %)	1 (0.5)	—	0 (0)	—	0 (0)	—	3 (0.3)
GWG (kg)	12.32 ± 1.34	—	13.12 ± 1.53	—	12.78 ± 1.51	—	12.64 ± 1.67
Infant birth weight (g)	3496.35 ± 552.14	< .001	3401.01 ± 426.48	—	3317.18 ± 382.87	—	3333.92 ± 400.57
LGA	56 (29.0)	< .001	9 (6.5)	—	5 (7.0)	—	69 (7.8)
The second trimester							
OGTT							
FBG (mmol/L)	5.20 ± 0.46	< .001	5.06 ± 0.47	< .001	5.04 ± 0.49	< .001	4.80 ± 0.44
1 h glucose OGTT (mmol/L)	9.32 ± 1.79	< .001	8.84 ± 1.80	.001	9.13 ± 1.78	< .001	8.09 ± 1.85
2 h glucose OGTT (mmol/L)	7.56 ± 1.39	< .001	7.58 ± 1.34	< .001	7.68 ± 1.62	.002	6.97 ± 1.40
AUC (glucose)	15.7 ± 1.24	< .001	15.16 ± 1.12	< .001	15.49 ± 1.45	< .001	13.98 ± 1.07
Fasting insulin (uU/mL)	16.00 ± 4.65	< .001	7.51 ± 2.59	—	10.74 ± 2.99	—	8.81 ± 3.69
1‐h insulin OGTT (uU/mL)	139.32 ± 41.23	< .001	46.26 ± 18.85	.041	49.43 ± 17.61	.046	61.30 ± 14.65
2‐h insulin OGTT (uU/mL)	147.34 ± 42.45	< .001	39.37 ± 19.31	.027	41.02 ± 14.87	.034	55.86 ± 16.28
AUC (insulin)	220.94 ± 40.23	< .001	69.75 ± 15.76	< .001	75.31 ± 11.23	< .001	93.64 ± 15.31
Insulin sensitivity (ISI composite index)	47.56 ± 14.54	< .001	159.26 ± 36.76	—	80.91 ± 17.34	.026	139.14 ± 43.75
Insulin secretion (Stumvoll I index)	1498.54 ± 301.78	< .001	664.45 ± 143.22	< .001	689.34 ± 188.56	< .001	944.32 ± 244.16

*Note*: Data are presented as *n* (%) for categorical variables, median (interquartile range) or mean (SD) for continuous variables. Differences across the three groups (NGT and two GDM subtypes) were compared using one‐way analysis of variance for normally distributed continuous variables, Kruskal–Wallis test for the skewed distributed continuous variables, or chi‐squared test for categorical variables. When *p* < .05, pairwise comparisons between the NGT group and each GDM subgroups were made using the Tukey's test, Dunn's test, or chi‐square test, respectively. *p* values for pairwise comparisons were adjusted using the Bonferroni correction.

Abbreviations: AUC, area under the curve; FBG, fasting blood glucose; GDM, gestational diabetes mellitus; GWG, gestational weight gain; ISI, insulin sensitivity index; LGA, large gestational age infant; NGT, normal glucose tolerance; OGTT, oral glucose tolerance test; pre‐BMI, pre‐pregnancy body mass index.

### 3.2 LGA risk prediction model based on logistic regression analysis

In our study, the training set (900 pregnant women) and the validation set (385 pregnant women) were randomly assigned from the cohort. The demographic and clinical characteristics of the two groups were shown in Table 2; no significant statistic differences were found between the two sets of the included variables.

**TABLE 2:** The clinical characteristics of the training cohort and the validation cohort.

Variables	Training cohort (*n* = 900)	Validation cohort (*n* = 385)	*p*
NGT (*n*, %)	616 (68.4)	267 (69.4)	.748
GDM‐resistance (*n*, %)	135 (15.0)	58 (15.1)	.976
Non GDM‐resistance (*n*, %)	146 (16.2)	63 (16.4)	.950
LGA (*n*, %)	97 (10.8)	42 (10.9)	.945
Maternal age (year)	30.9 ± 3.8	30.5 ± 4.0	.561
T2DM family history (*n*, %)	84 (9.3)	30 (7.8)	.373
pre‐BMI (kg/m^2^)	21.9 ± 3.2	21.9 ± 3.0	.099
Age at menarche ≤11 years (*n*, %)	66 (7.3)	18 (4.7)	.077
ART (*n*, %)	61 (6.7)	23 (6.0)	.593
Thyroid antibodies + (TPOAb/TgAb)	129 (14.3)	44 (11.4)	.162
Above IOM recommended GWG	169 (18.7)	76 (19.7)	.687
History of macrosomia	30 (3.3)	12 (3.1)	.842
Parity	1.30 (0.66)	1.33 (0.63)	.190
Vitamin B_12_ (pg/mL)	58.99 (37.21–82.54)	65.84 (51.93–81.84)	.194
Ferritin (ng/mL)	45.65 ± 37.21	43.72 ± 37.21	.303
Total protein (g/L)	69.33 (65.21–74.91)	69.03 (64.88–74.18)	.851
Albumin (g/L)	43.11 ± 8.45	40.11 ± 7.87	.203
Globulin (g/L)	29.28 ± 3.33	28.73 ± 3.06	.280
ALT (U/L)	18.12 ± 3.12	18.18 ± 2.4	.924
AST (U/L)	19.18 ± 4.76	18.80 ± 5.12	.537
CHO (mmol/L)	6.1 ± 1.2	6.1 ± 1.1	.701
TG (mmol/L)	3.6 ± 1.6	3.6 ± 1.6	.568
HDL‐C (mmol/L)	1.8 ± 0.4	1.8 ± 0.4	.524
LDL‐C (mmol/L)	3.1 ± 0.8	3.2 ± 0.9	.392
FBG (mmol/L)	4.3 ± 0.8	4.4 ± 0.7	.136

*Note*: Data are presented as *n* (%) for categorical variables, median (interquartile range) or mean (SD) for continuous variables. Differences between the two groups were analyzed by the *t* test and the Mann–Whitney U test for the normally distributed continuous variables and the skewed distributed continuous variables, Chi‐square test was employed to analysis the categorical variables.

Abbreviations: ALT, alanine aminotransferase; ART, assisted reproductive technology; AST, aspartate aminotransferase; CHO, total cholesterol; FBG, fasting blood glucose; GDM, gestational diabetes mellitus; HDL‐C, high‐density lipoprotein cholesterol; IOM, Institute of Medicine; LDL‐C, low‐density lipoprotein cholesterol; NGT, normal glucose tolerance; pre‐BMI, pre‐pregnancy body mass index; T2DM, type 2 diabetes mellitus; TG, triglyceride.

### 3.3 LASSO logistic regression and cross‐validation of LASSO logistic regression for the selection of variables

**FIGURE 1:**
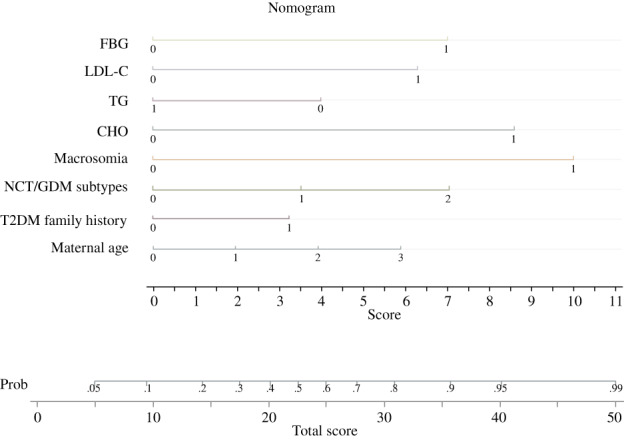
Nomogram for predicting LGA risk. The total score is calculated by the sum score of FBG, LDL‐C, TG, CHO, pregnancy history of macrosomia, FBG (1 if ≥5.3 mmol/L and 0 if <5.3 mmol/L), LDL‐C (1 if ≥4.92 mmol/L and 0 if <4.92 mmol/L), TG (1 if ≥4.63 mmol/L and 0 if <4.63 mmol/L), CHO (1 if ≥8.2 mmol/L and 0 if <8.2 mmol/L), macrosomia (0 if no and 1 if yes), NGT/GDM subtypes (0 if NGT, −0.367 if the GDM nonresistance subtype, 1.294 if the GDM‐resistance subtype), T2DM family history (0 if no and 1 if yes), and maternal age (0 if <30, 1 if 30–34, 2 if 35–39, 3 if ≥40). CHO, cholesterol; FBG, fasting blood glucose; GDM, gestational diabetes mellitus; LDL‐C, low density lipoprotein cholesterol; LGA, large for gestational age; NGT, normal glucose tolerance; T2DM, type 2 diabetes mellitus; TG, triglyceride.

The LASSO logistic regression analysis and cross‐validation were employed to select the variables (Figure 1 in Appendix S1). Maternal age stratification, type 2 diabetes mellitus (T2DM) family history, GDM subtypes, macrosomia history, age at menarche ≤11 years, CHO, TG, HCL‐C, LDL‐C, and FBG were screened out as variables that constitute the final model.

We employed multivariable logistic regression analysis to calculate the relevant β‐coefficients and constant based on the 10 variables screened out by LASSO logistic regression. The final multivariate logistic regression prediction model was developed by eight variables based on the backward step algorithm (Table 3). The predicted LGA risk is estimated by the equation:

$$P=\frac{1}{1+\exp.\left(-x\right)},X=-3.162+0.611\;\text{Maternal}\;\mathrm{Age}\;30-34y/0.920\;\text{Maternal}\;\mathrm{Age}\;35-39y/0.683\;\text{Maternal}\;\mathrm{Age}\geq 40y+0.606\;T2\mathrm{DM}\;\text{family history}–0.367\;\mathrm{GDM}\;\text{nonresistance}/+1.294\;\mathrm{GDM}\;\text{resistance}+1.580\;\text{macrosomia}+1.522\;\mathrm{CHO}-0.679\;\mathrm{TG}+1.104\;\mathrm{LDL}‐C+1.151\mathrm{FBG}.$$

**TABLE 3:** The development of model by multivariate logistic regression.

Variables	B	SE	Wald	*p*	OR	95% CI
Maternal age <30 years (reference)			7.634	.054		
30–34 years	0.611	0.287	4.537	.033	1.843	1.050–3.233
35–39 years	0.920	0.356	6.684	.010	2.510	1.249–5.043
≥40 years	0.683	0.720	0.900	.343	1.979	0.483–8.109
T2DM family history	0.606	0.347	3.055	.081	1.833	0.929–3.615
NGT (reference)			27.025	<.001		
GDM nonresistance	−0.367	0.376	0.954	.329	0.692	0.331–1.447
GDM resistance	1.294	0.280	21.356	<.001	3.648	2.107–6.315
macrosomia	1.580	0.479	10.902	<.001	4.856	1.901–12.408
CHO	1.522	0.443	11.819	.001	4.580	1.924–10.905
TG	−0.679	0.355	3.656	.056	0.507	0.253–1.017
LDL‐C	1.104	0.518	4.544	.033	3.017	1.093–8.329
FBG	1.151	0.360	10.246	1	0.001	3.161–1.562
Constant	−3.162	0.261	146.958	1	0.000	0.042

Abbreviations: CHO, total cholesterol; CI, confidence interval; FBG, fasting blood glucose; GDM, gestational diabetes mellitus; LDL‐C, low‐density lipoprotein cholesterol; LGA, large for gestational age; NGT, normal glucose tolerance; OR, odds ratio; T2DM, type 2 diabetes mellitus; TG, triglyceride.

### 3.4 Nomograph

We employed nomogram to visualize and popularize the LGA prediction model (Figure 1).

### 3.5 Validation of the prediction model

The AUC for the training set is 0.760 (95% CI 0.706–0.815), and 0.748 (95% CI 0.659–0.837) for the validation set (Figure 2A,D in Appendix S1), which suggested a good discriminative capacity of the LGA prediction model. The calibration curves of the model showed that the result of the training set and the validation set corresponded well (Figure 2B,E in Appendix S1). The DCA plot was shown in Figure 2C,F in Appendix S1, which indicated certain positive net benefits in the prediction model among majority threshold probabilities. The Hosmer–Lemeshow test demonstrated a nonsignificant statistical difference in each set (All *p* > .05, Table 4), which suggested a high consistency. The generalization of the logistic regression model is presented in Table 5.

**TABLE 4:** Hosmer and Lemeshow test.

Set	Chi‐square	Sig.
Training set	6.346	0.500
Validation set	5.119	0.529

**TABLE 5:** The generalization of the models.

Models	AUC _Training set_ (95% CI)	AUC _Validation set_ (95% CI)	Precision	Recall	F1‐score	Accuracy	Specificity
Logistic regression model	0.760 (0.706–0.815)	0.748 (0.659–0.837)	0.638	0.600	0.618	0.641	0.680
Decision tree	0.813 (0.786–0.839)	0.779 (0.735–0.824)	0.672	0.668	0.670	0.696	0.719
Random forest	0.854 (0.831–0.877)	0.808 (0.766–0.850)	0.707	0.695	0.701	0.725	0.751

Abbreviations: AUC, area under the curve; CI, confidence interval.

### 3.6 Machine learning models of the LGA prediction model

Figure 3 in Appendix S1 shows receiver operating characteristic curves of the training set and validation set of the DT model and the RF model respectively. Spearman correlation coefficient and Pearson correlation coefficient were employed to sort all variables relative to LGA group and normal birth weight group for the two ML models (Figure 4 in Appendix S1). Figure 5 in Appendix S1 and 6 show the tree structure of the two ML models. The generalization of the two ML models is presented in Table 5. Our ML models achieved an expected discrimination.

## 4 DISCUSSION

We established three LGA prediction models that could be used at an early stage of the third trimester by the logistic regression analysis (eight variables) and the two ML algorithms (DT and RF, all variables). The AUCs for the training sets and validation sets are 0.760 (95% CI 0.706–0.815) and 0.748 (95% CI 0.659–0.837) for the logistic regression analysis model, 0.813 (95% CI 0.786–0.839) and 0.779 (95% CI 0.735–0.824) for the DT model, 0.854 (95% CI 0.831–0.877) and 0.808 (95% CI 0.766–0.850) for the RF model.

Women of GDM subtypes inclined to have LGA infants might be caused by different mechanisms.^6^, ^7^ Women with GDM‐resistance showed a character of relatively higher pre‐BMI than the NGT group.^7^, ^20^ Previous study established the LGA prediction model including GDM subtypes as a variable.^21^ However, the model showed a limited prediction power with an AUC of 0.698 for the training set, which may be related to the following reasons: the variables in the model were all in without screened by LASSO regression; the diagnosis criteria of GDM was based on the results of OGTT at the 24th–32th gestational week ranged by different regions, rather than the OGTT at the 24th–28th gestational week recommended by IADPSG; and the effect of plasma lipid profile at late pregnancy on LGA was not considered. Lene and colleagues^22^ found that the combination of Matsuda index (insulin sensitivity) and their disposition index (DI) significantly increased the prediction power of LGA than the GDM subtypes; however, DI is calculated as Matsuda index multiplied by Stumvoll I index (insulin secretions), which amplified the weight of Matsuda index and without the consideration of collinearity between the two continuous variables. At the same time, as a multicenter study, Lene and colleagues^22^ classified the GDM subtypes based on the summarized data of all centers worldwide to calculate the normal range of insulin sensitivity and insulin secretion indexes, without taking into account the existing differences caused by race.

Maternal overweight/obesity is closely related to the birth weight of newborns.^23^, ^24^ However, pre‐BMI stratification was not included in the LASSO regression analysis in our logistic regression model. We speculate that it might be related to the obesity character of the GDM‐resistance subtype, which leads to the limited importance of pre‐BMI in the logistic regression model. Actually, we combined the influence of insulin sensitivity/secretions and obesity as GDM subtypes, which reflect the whole pathophysiological process leading to the elevated blood glucose during pregnancy.

Modifiable risk factors are essential to identify and remeasure the relationship of the risk factor and outcomes, which is important for prevention. Maternal FBG and lipid profile at the early stage of the third trimester were also considered as two modifiable independent variables in the published LGA prediction model,^25^, ^26^ which leaves enough time for diet and exercise interventions before delivery. Maternal lipid profile of TG, HDL‐C, and LDL‐C were increased within a physiological range from the second to third trimester during pregnancy^27^; however, an independent relationship emerged between overrange lipid profile and fetal overgrowth,^10^ including the level of TG at late pregnancy.^25^ A prospective study showed that the level of maternal fasting and postprandial TG manifested a stronger power in predicting neonatal fat content than FBG after strict glycemic control.^28^ According to the TSRIs for blood lipid profiles in Chinese pregnant women,^19^ the levels of FBG, CHO, TG, HDL‐C, and LDL‐C at the 28th–32th gestational week above 5.3 mmol/L, 8.2 mmol/L, 4.63 mmol/L, 1.24 mmol/L, and 4.92 mmol/L are associated with adverse pregnancy outcomes. However, studies on the effect of improving maternal lipid profiles at the 3rd trimester on fetal outcomes are limited.

The relationship between maternal dyslipidemia and fetal overgrowth is mediated by placenta lipid transport capacities.^29^ Lipases in placental microvillous membrane could hydrolyze plasma lipoproteins, such as TG, phospholipids, and other lipid nutrients in maternal circulation, and release to nonesterized fatty acid (NEFA). The translocation of NEFA in the placenta is through simple diffusion of fatty acid transporter proteins, fatty acid translocases, fatty acid binding proteins, and major facilitator superfamily domain containing protein2a in a transporter‐controlled manner.^30^, ^31^ NEFAs can cross the base membrane into the fetal circulation and then be absorbed by the fetal liver and eventually cause fetal overgrowth.^32^ Our previous study found that elevated placental lipid content (TG, CHO) at the third trimester of overweight pregnant women enhanced placental mTORC1‐RPS6 and ERK1/2 signaling, leading to the rise of cord blood insulin level and birth weight.^11^

The relationship between previous macrosomia delivery history and the risk of having LGA might be caused by diet and the awareness of monitoring fetal overgrowth at the third trimester. T2DM and GDM have many overlapped susceptible genes.^33^, ^34^ The function of islet cells decreased accompanied with the increasing of maternal age.^35^ Accompanied by advanced maternal age, pregnancy increased the metabolic load of mother, revealed small defects (associated with T2DM genes) of islet cell for the mother. Finally, leading to the elevated blood glucose during pregnancy, and increases the risk of fetal overgrowth.

For the comparison among the three prediction models, the logistic regression model including eight common variables and the formula/nomograph could be used for external verification. By contrast, the two ML models including all variables are difficult to popularize due to the algorithm limitation. As for ML, the prediction models established by DT and RF show relatively good performance in the validation set, with improved receiver operating characteristics, accuracy, sensitivity, and specificity relative to the traditional logistic model, which is due to the advanced algorithms of ML. In details, DT and RT have certain features of selection ability, which can show the ranking of feature importance intuitively; DT has certain interpretability, and the structure of the tree can be visualized; RF has the ability to prevent overfitting, and the performance of accuracy is better than most single algorithms. However, ML models still have shortcomings. To be specific, the algorithm of some ML is a black box, which cannot be reflected by concise diagrams or formulas. In addition, ML models are difficult to popularize than logistic models due to the high requirements on computing power and computing environment.

The advantages of our research include that the definition of LGA is based on the distribution of birth weight corresponding to the sex of newborn. It has reported that the combination of predictors including glycemic measures, BMI, and maternal age showed an increased power in predicting LGA than individual indicators,^36^, ^37^ and our LGA prediction model has taken all these indicators into account. The continuous variables in our model were transformed into dichotomous variables according to the reported target during pregnancy, avoiding collinearity among variables. Modifiable variables were included in our models, which can be used to access the remission after some interventions.

The limitations of our study include a relatively small sample size and single‐center study, which limited the further adjustment for ethnicity differences and other confounding factors.

In conclusion, we established three LGA prediction models that can be used at early stage of the third trimeste to identify pregnant women with a high risk of giving birth to LGA and to guide prevention strategies at an early stage of the third trimester.

## AUTHOR CONTRIBUTIONS

Ning Wang, Lin Song, and Wei Cui designed the work presented by the article. Ning Wang, Haonan Guo, and Yingyu Jing completed the meta‐analysis and drafted and revised the article. Bo Sun, JJing Xu, Huan Chen, and Mengjun Wang collected the data and revised the article for critically important content. Lin Song and Wei Cui final approved of the version to be published. All authors contributed to the article and approved the submitted version.

## FUNDING INFORMATION

We acknowledge grant funding of the Natural Science Foundation of Shaanxi Province (No. 2020GXLH‐Y‐029, 2019JQ069, 2019JM262), the Natural Science Foundation of China (No. 81801459; No. 82071732; No. 81741079), the Natural Science Foundation for Postdoctoral Scientists of China (No. 2018M641001, No. 2016M600799), the Clinical Research Award of the First Affiliated Hospital of Xi'an Jiaotong University, China (No. XJTU1AF‐CRF‐2019‐007).

## CONFLICT OF INTEREST STATEMENT

The authors declare no potential conflicts of interest.

## CONSENT TO PARTICIPATE

Individuals were informed about the use of their data and were offered an opt‐out. All of the included participants gave their informed consent. The data were used anonymously. Written informed consent was obtained from the parents.

## Supporting information


**Appendix S1.** Supporting information

## Data Availability

The data associated with the paper are not publicly available but are available from the corresponding author on reasonable request.
