# Supplementary material for: Development and validation of risk prediction models for large for gestational age infants using logistic regression and two machine learning algorithms
Source: J Diabetes. 2023 Mar 8;15(4):338–48. doi: 10.1111/1753-0407.13375 (PMC10101839; doi:10.1111/1753-0407.13375)

**Supplementary Figure 1.** The selection method of the variables.


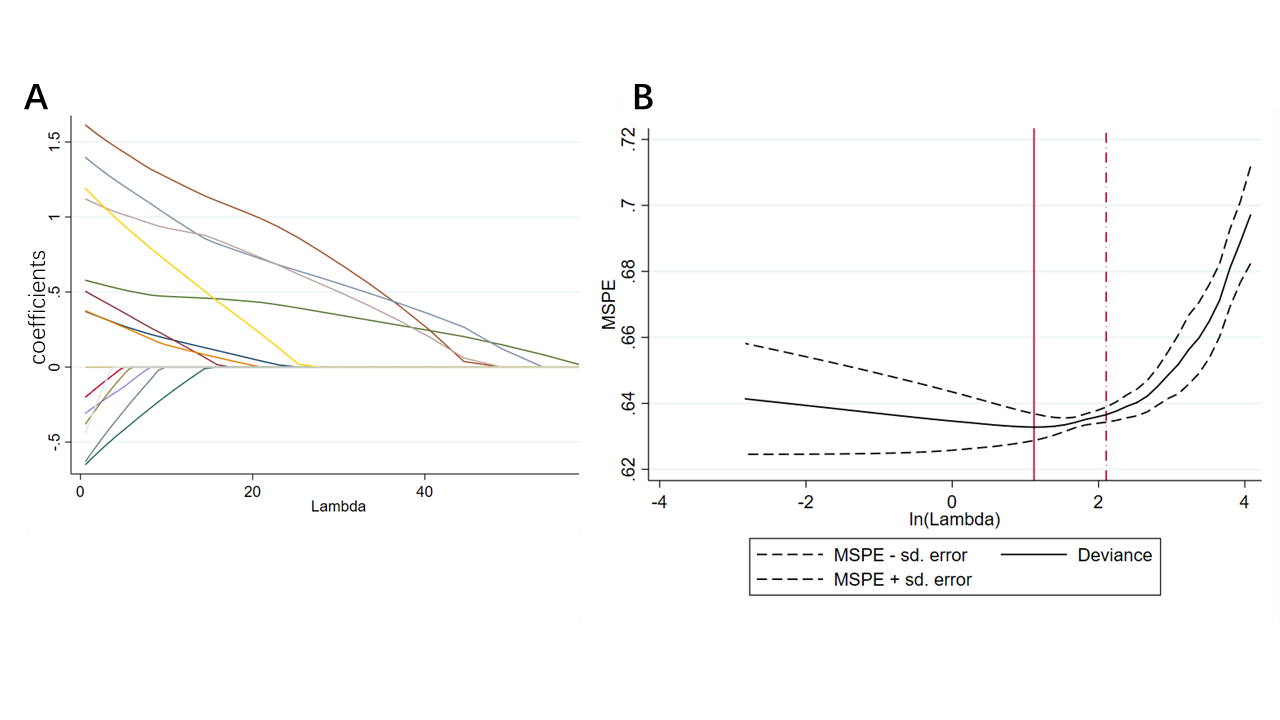


(A)Variables selected by the LASSO regression analysis. (B) Selection of the parameter (Lambda) in LASSO applied tenfold cross-validation via minimum criteria. Lambda was based on the minimum criteria (the left solid line) and one standard error of the minimum criteria to adjust deviations.

**Supplementary Figure 2.** ROC curve graphs, the calibration curve and the decision curve analysis of the training set and the validation set.


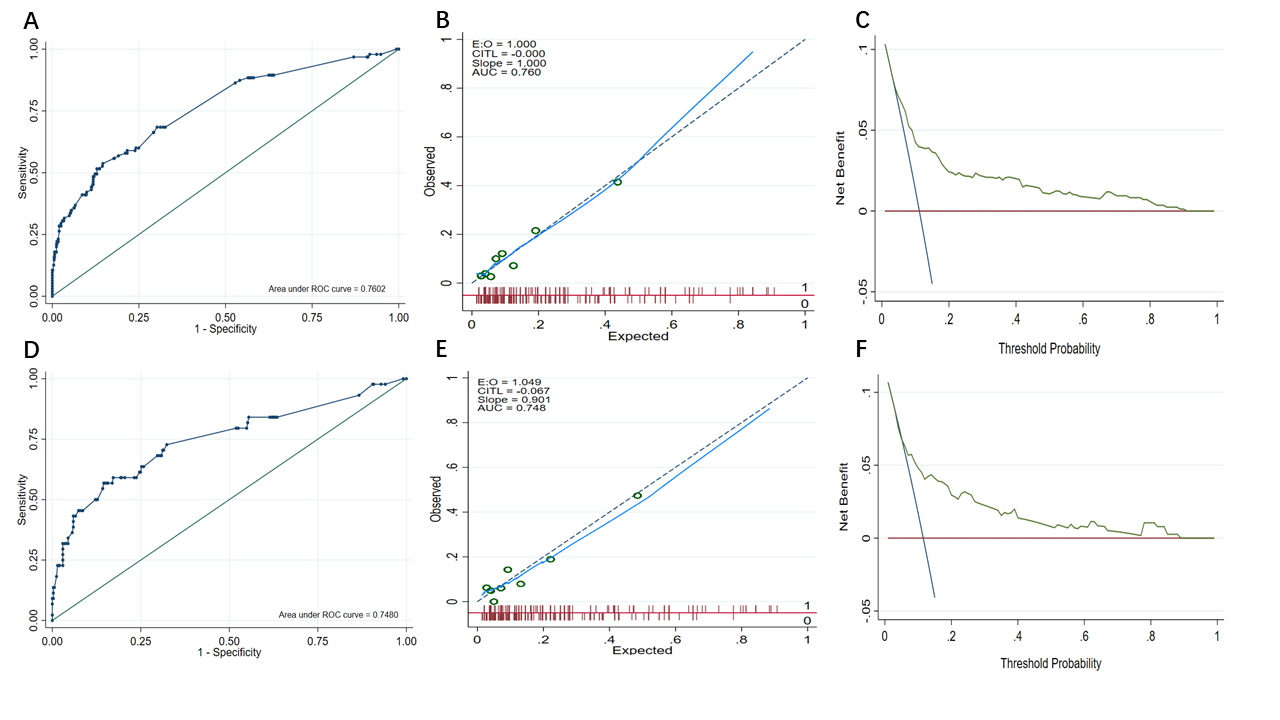
ROC curves for the training set (A) and the validation set (D). Calibration curves for the training set (B) and the validation set (E). DCA curve for the training set (C) and the validation set (F).

**Supplementary Figure 3.** ROC curves of the training set and the validation set by decision tree model and the random forest model.


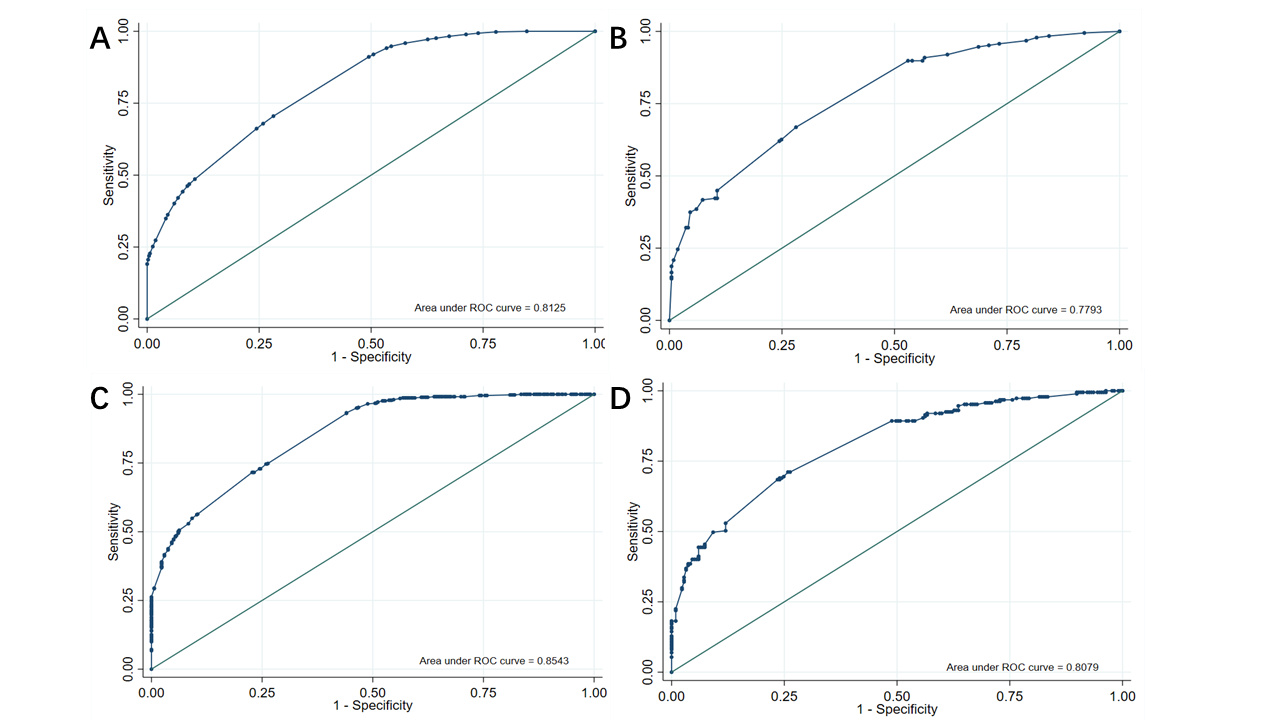


(A) is the ROC curve for the training set, and (B) for the validation set of the Decision tree model. For the Random Forest model, (C) is for the training set, and (D) for the validation set.

**Supplementary Figure 4.** Correlations between features and class importance.


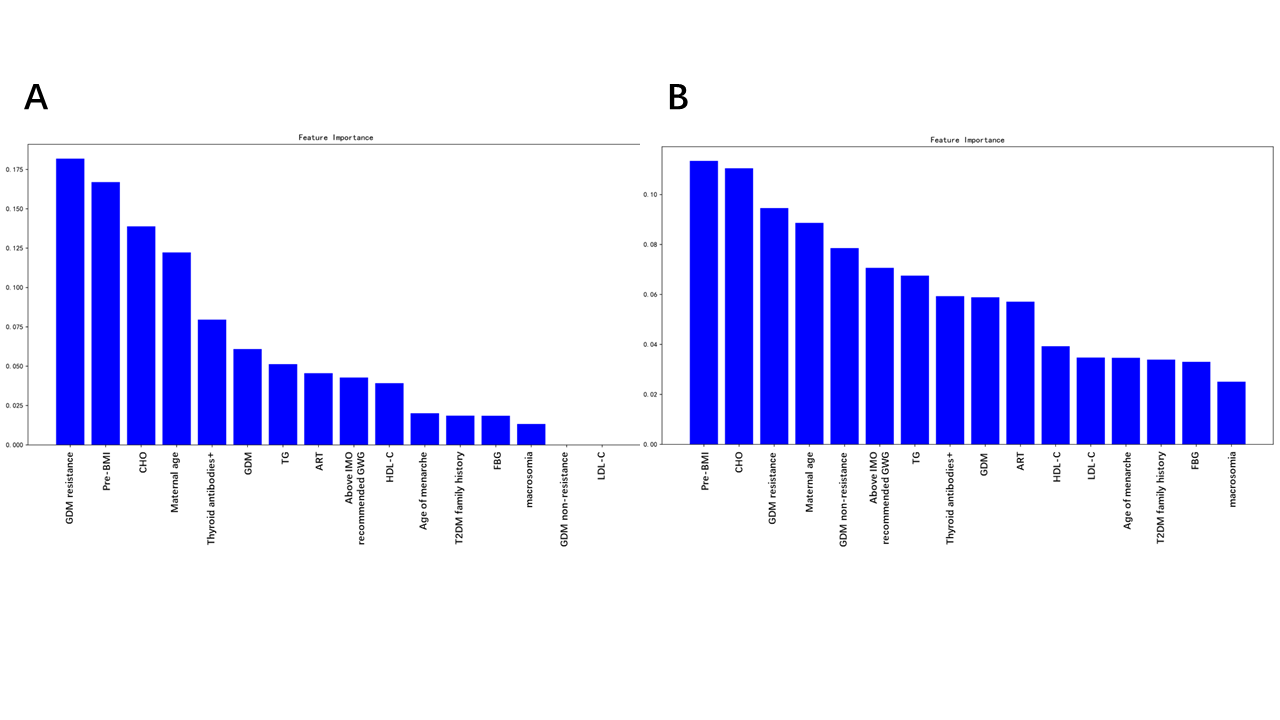


Spearman correlation coefficients of variables and the Larger Gestational Age (LGA) /non-LGA label vector, over all the samples. The absolute values from high to low are represented by the bar plots from left to right. (A) is for the decision tree, and (B) is for the random forest.

**Supplementary Figure 5.** The tree structure of decision tree model.


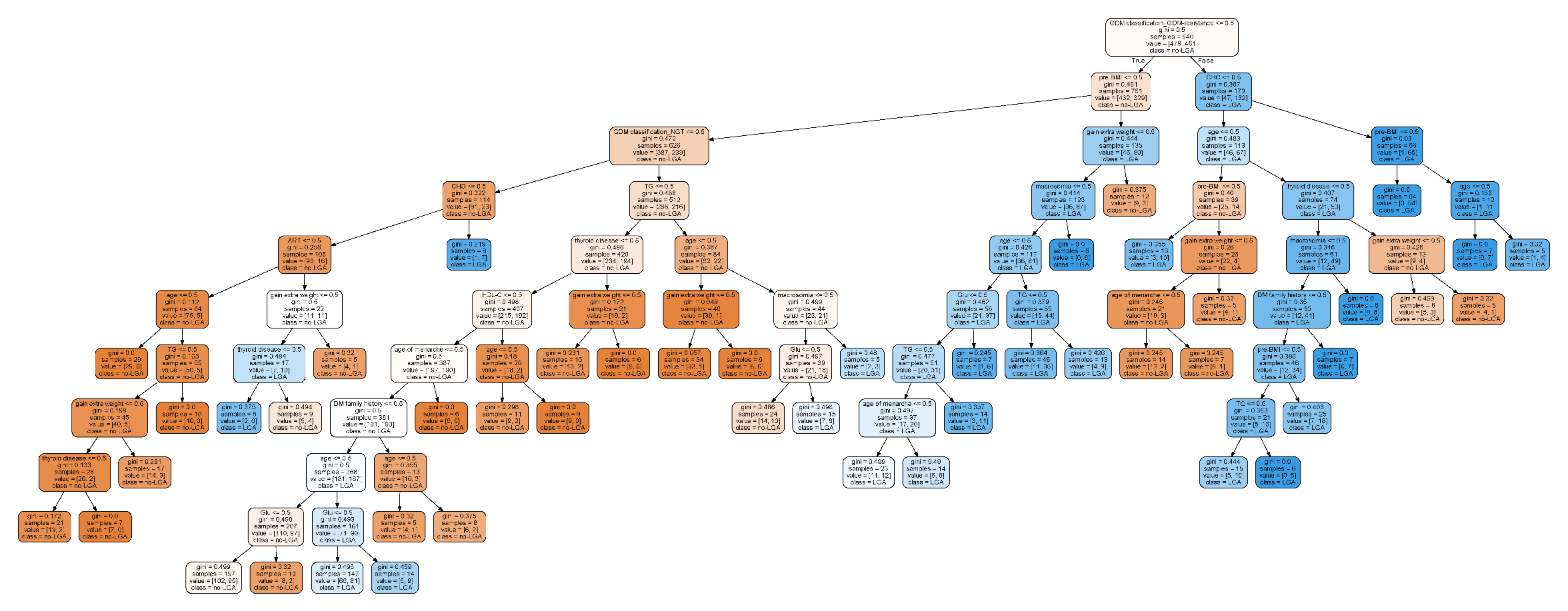


**Supplementary Figure 6.** The tree structure of random forest model.


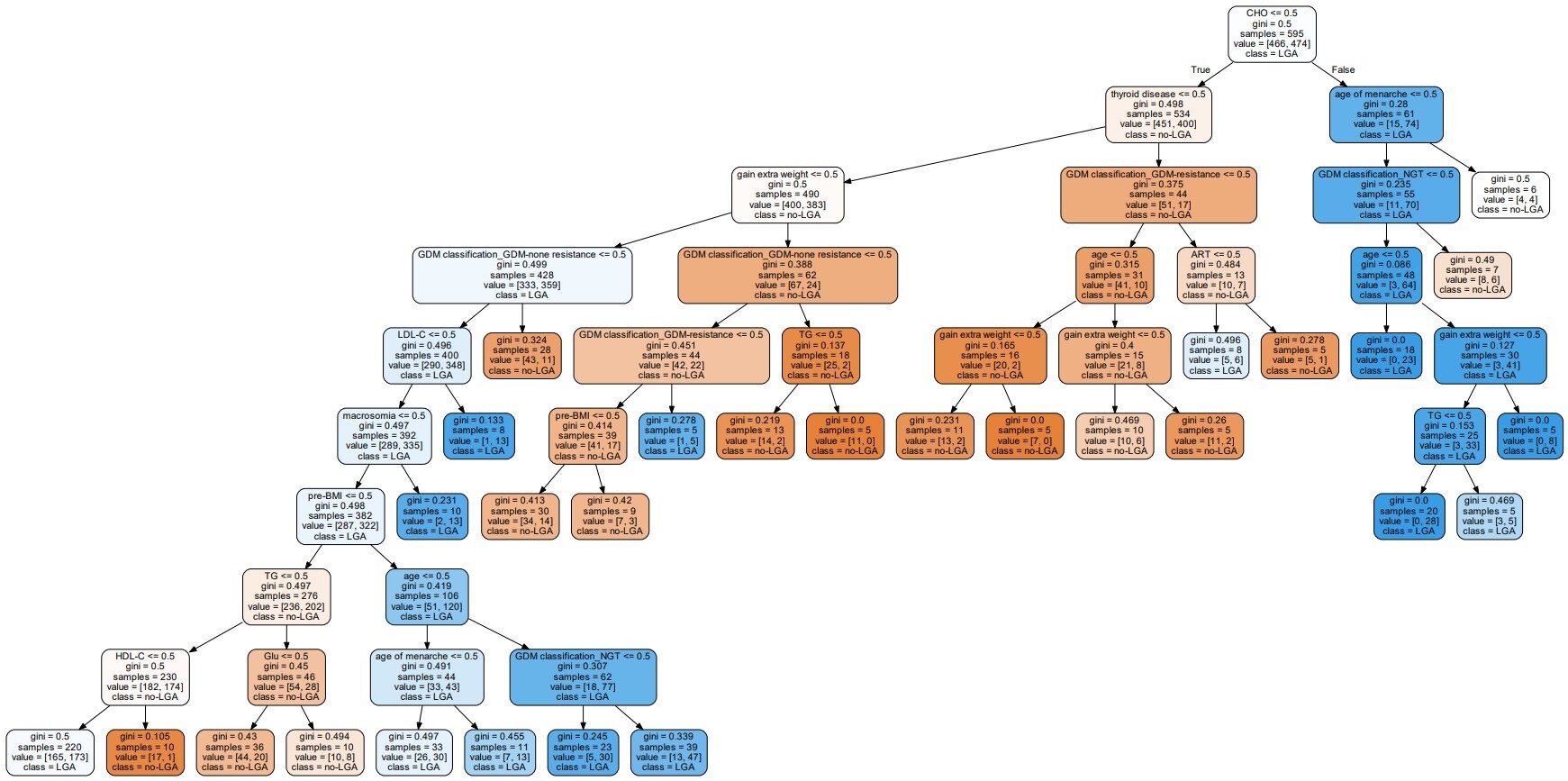

Supplement: Supplementary file 1 — Appendix S1. Supporting information [file JDB-15-338-s001.docx]
